# Uptake of Travel Health Services by Community Pharmacies and Patients Following Pharmacist Immunization Scope Expansion in Ontario, Canada

**DOI:** 10.3390/pharmacy7020035

**Published:** 2019-04-13

**Authors:** Sherilyn K. D. Houle, Kristina Kozlovsky, Heidi V. J. Fernandes, Zahava Rosenberg-Yunger

**Affiliations:** 1 School of Pharmacy, University of Waterloo, Waterloo, ON N2L 3G1, Canada; k.kozlovsky@hotmail.com (K.K); heidi.fernandes@uwaterloo.ca (H.V.J.F.); 2 Ted Rogers School of Management, Ryerson University, Toronto, ON M5G 2C3, Canada; zahava.rosenberg@ryerson.ca

**Keywords:** travel, immunization, vaccination, pharmacist, community pharmacy

## Abstract

In December 2016, pharmacists in Ontario, Canada with authorization to administer injections saw an expansion in their scope from a restriction to the influenza vaccination only to now including an additional 13 vaccine-preventable diseases, largely those related to travel. It was uncertain whether this change in scope would see sufficient uptake, or translate to a corresponding expansion in other travel health service offerings from community pharmacies. In October/November 2017 a survey was conducted of all licensed community pharmacists in Ontario, followed by semi-structured interviews with 6 survey respondents in June 2018. A web-based survey of members of the public from a single region of the province was also conducted in September 2018 to assess uptake of expanded vaccination services. Broad variability in uptake of these services was noted, ranging from the dispensing of travel-related medications and vaccinations only through to vaccine administration and prescribing under medical directive; however, uptake was generally at the lower end of this spectrum. This was evidenced by 94% of pharmacists reporting administering fewer than 10 travel vaccinations per month, fewer than 10% of patients reporting receiving a travel vaccine administered by a pharmacist, and a maximum of 30 pharmacies (of nearly 6000 in the province) designated to provide yellow fever vaccinations. Fewer than 1 in 3 pharmacists reported performing some form of pre-travel consultation in their practice, often limited to low-risk cases only. Barriers and facilitators reported were similar for these services as they were for other non-dispensing services, including insufficient time to integrate the service into their workload, perceived lack of knowledge and confidence in travel health, and low patient awareness of these new services available to them through community pharmacies.

## 1. Introduction

In 2012, pharmacists in Ontario, Canada, were authorized to administer the influenza vaccine to patients age 5 years and older following the successful completion of an immunization training program, and obtaining valid certification in cardiopulmonary resuscitation and first aid [1]. In December 2016, amendments were made to the Pharmacy Act to permit injection-trained pharmacists, pharmacy students, and interns to administer vaccines to patients for 13 travel and travel-related vaccine-preventable diseases [2], listed in Table 1. Of note, this legislation only authorizes the administration of each vaccine, with some still requiring a prescription as indicated in the table [3]. Pharmacists in Ontario currently do not have broad prescribing authorization for vaccines, although pharmacists can pursue medical directives from physicians or nurse practitioners to prescribe these vaccines under delegation [4]. Vaccines currently on Ontario’s routine immunization schedule [5] can be obtained at no charge from a physician office or public health clinic; however, any of these vaccines received in a pharmacy must be paid for by the patient, as well as any administration fee that may be charged by the pharmacy. Some patients may be eligible for coverage through private insurance, although this varies by insurer and by vaccine.

This expansion in scope may have a positive impact on the health of travellers by offering additional points of access to vaccinations and other travel-related care. Indeed, the convenience of pharmacist-administered vaccinations has been cited as a key factor influencing patients’ decisions to be vaccinated at a pharmacy [6]. One study reported that 30% of vaccinations administered by a U.S. pharmacy chain were administered outside of usual physician office hours, including weekday evenings, weekends, and national holidays [7]. However, vaccinations are only one component of travel-related risk reduction, with infectious diseases representing less than 2% of deaths among travelers [8]. However, seeking necessary vaccinations can be a driver for travelers to seek pre-travel healthcare advice. This creates the opportunity for clinicians to assess patient-, itinerary-, and destination-specific risks, as well as prophylactic drug therapy and education on non-drug health and safety measures [9].

There is evidence on the potential positive impact that pharmacists can have on patients’ travel health care. With respect to vaccinations, previous studies have reported higher vaccination rates against seasonal influenza in jurisdictions that authorize pharmacists to administer vaccinations [10,11]. It is uncertain whether a similar trend is observed with travel vaccines. There is also evidence on the clinical outcomes achievable from pharmacist-performed travel consultations, including high acceptance rates of recommended vaccinations and medications, low incidence of illness while travelling, and patient confidence in their ability to self-manage illness abroad [12,13]. Finally, pharmacist-provided services were observed to lead to significant cost savings in one study as a result of lower use of unnecessary vaccinations and medicines when compared to consultations performed in a nurse-based travel service. This study also reported that pharmacists were able to identify and order missing vaccinations that were indicated for patients outside of just travel vaccinations (e.g., pneumococcal vaccine) [14]. Many of these studies were conducted among pharmacists with a specialized practice and additional training in travel health; moreover, other work has suggested that the general population of community pharmacists lack confidence in their ability to perform pre-travel consultations and assess the appropriateness of prescribed travel vaccines [15,16].

It is unknown whether an expansion in pharmacists’ scope to include the administration of vaccines for travel-related purposes would translate to expanded offerings of multifaceted travel health services in community pharmacies, or if pharmacists would embrace this new scope in addition to their existing workload. The objective of this study is to examine data available to date on the landscape of travel health services in Ontario community pharmacies following this change in scope, pharmacists’ perceptions of their ability to provide these services in practice, and patients’ self-reported receipt of these additional vaccines by pharmacists, to determine the initial impact of this legislative change on pharmacy practice and patient care.

## 2. Materials and Methods 

Triangulation of quantitative and qualitative data from four sources was performed:

### 2.1. Pharmacist Survey

Following a literature review, an online survey consisting of 38 questions was developed in consultation with a pharmacy practice researcher and an individual with expertise in health policy, and pilot tested for understandability and functionality among a sample of five third-year pharmacy students. The complete list of survey items is available in Appendix A. Questions sought basic demographic information about respondents, and explored the respondents’ current travel health service offerings, changes around service offerings since the expansion in scope of December 2016, barriers and facilitators to service implementation, and further educational needs. Inclusion criteria for the survey were: (1) Current practice in a community pharmacy; and (2) Part A (active practice) licensure through the Ontario College of Pharmacists. The survey was administered using Qualtrics^TM^ software (Qualtrics, Provo, UT, USA) and disseminated through the following methods: the Ontario Pharmacists Association’s weekly email bulletins, social media, direct contact with known pharmacists, and through email to all Part A pharmacists in Ontario who provided permission to the Ontario College of Pharmacists to have their contact information utilized for research. Descriptive statistics were performed using IBM SPSS Statistics for Mac, Version 25 (Armonk, NY, USA). Ethics approval was received from the University of Waterloo Research Ethics Committee (ORE #22510) and Ryerson University’s Research Ethics Board (REB 2018-282).

### 2.2. Pharmacist Interviews

At the conclusion of the pharmacist survey described above, respondents were asked to express interest in being contacted for a follow-up interview to gain deeper insight into reasons contributing to variable uptake across pharmacists and pharmacies, and to identify potential strategies to provide necessary support to pharmacists who wish to expand their patient care services in this area. Interested individuals were contacted and asked to participate in a telephone interview at a mutually agreeable time. A semi-structured interview guide was developed and is available in Appendix B. No compensation was provided for participants. Interviews were performed until data saturation was achieved, based on the perspective of the interviewer (JPS) and the principal investigator (SH). Interviews were audio-recorded and transcribed verbatim, with qualitative content analysis performed independently by two team members (ZRY and HF) for the first three transcripts to identify and summarize themes, and any disagreements resolved by consensus. The remaining transcripts were coded by one team member (HF). Analysis was performed using Microsoft Excel for Mac, version 15.33. Ethics approval was received from the University of Waterloo Research Ethics Committee (ORE #31442) and the Ryerson University Research Ethics Board (REB 2018–282). 

### 2.3. Public Survey

Annually, the Survey Research Centre at the University of Waterloo conducts a Waterloo Regional Area Web Panel Survey. This omnibus survey allows researchers to pool resources for surveying the general public residing in the Waterloo region of Ontario, Canada. As such, the survey addresses a variety of topics of relevance to the general public. Members of the panel were identified through random digit dialing using both landline and cellular telephone numbers, followed by random selection of one member of the household for inclusion. Participants were then asked if they would be willing to be contacted by email to complete a web survey. Those who consent were invited by email to complete the survey, with two reminder emails at weekly intervals. Ethics approval for the survey was received from the University of Waterloo Research Ethics Committee (ORE #23309).

The 2018 survey included demographic questions such as employment status, household income, level of education, household size, and marital status, as well as a series of questions about receipt of pharmacist-administered vaccinations. Specifically, respondents were asked to indicate which (if any) vaccines they had ever received from a pharmacist. Each vaccine currently within the scope of practice for Ontario pharmacists was included. Web survey questions are provided in Appendix C.

Survey results were first analyzed descriptively using IBM SPSS Statistics for Mac, Version 25 (Armonk, NY, USA). Binary logistic regression was then performed to identify any associations between each demographic variable as independent variables and the receipt of any vaccine from a pharmacist, as well as each vaccine individually as the dependent variable. Statistical significance was defined a priori as *p* < 0.05.

### 2.4. Designated Yellow Fever Vaccination Centres

In Canada, the yellow fever vaccine is only distributed to healthcare facilities with current status as a Designated Yellow Fever Vaccination Centre through the Public Health Agency of Canada [17]. Pharmacies can obtain this designation, provided that they have either a pharmacist with authorization to prescribe the vaccine or have identified an individual with prescribing authority for the vaccine to serve as the site’s nominated healthcare practitioner. This individual is responsible for overseeing operations to ensure compliance with designated site requirements. As yellow fever is not endemic in Canada [18], the use of its vaccine here is limited to international travellers. Additionally, given the complexity related to its use and other travel health risks associated with travel to yellow fever endemic countries, it can be assumed that only those sites providing pre-travel consultations and travel vaccinations on a regular basis would apply for designation. As such, the number of Designated Yellow Fever Vaccination Centers over time can serve as a proxy for the provision of travel health services.

All sites with this designation are publicly listed online, and searchable by province; however, the Public Health Agency of Canada does not retain historical records on the number and type of sites. To identify changes in the number of designated sites in Ontario over time, lists of all designated sites in the province of Ontario [19] were generated at 3-month intervals, beginning with March 2017. Pharmacies on this list were identified by cross-referencing pharmacy names and addresses with the list of licensed pharmacies maintained by the Ontario College of Pharmacists [20]. All other sites were coded as one of the following: travel clinic, medical clinic (includes interdisciplinary Family Health Teams and urgent care centers), public health unit, corporate or occupational health clinic, or other. Facilities whose categorization were uncertain based on their name alone were identified by performing an internet search to visit the website of the organization and determining the most applicable category. In the event that a facility was listed as a ‘medical and travel clinic’ it was coded as a travel clinic with the assumption that travel consultations performed here would be more representative of those performed at a specialized travel clinic than a general medicine practice.

As this analysis uses publicly available data, research ethics approval is not required. Descriptive statistics were performed using IBM SPSS Statistics for Mac, Version 25 (Armonk, NY, USA).

## 3. Results

### 3.1. Pharmacist Survey

Of the 222 respondents initiating the survey, 17 were excluded for failing to provide consent to participate (n = 5), not currently working in a community pharmacy (n = 11), or not currently holding Part A licensure (n = 1). The demographic characteristics of the 205 respondents completing the survey is provided in Table 2.

**Figure 1:**
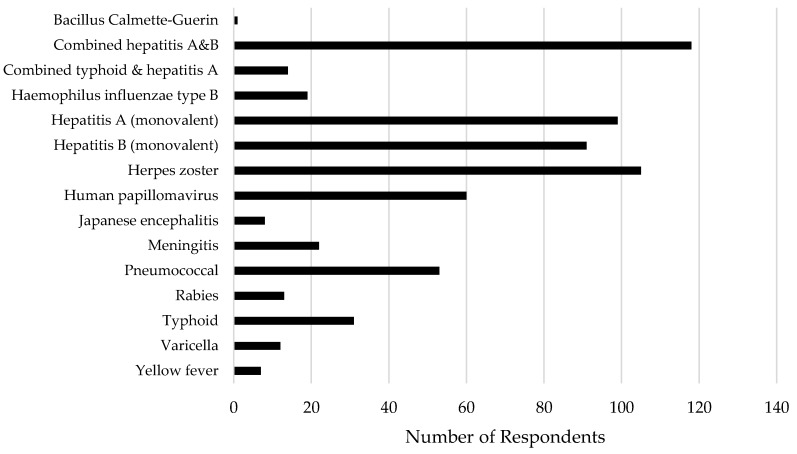
Pharmacist respondents’ self-report of vaccinations they have personally administered to patients since December 2016.

Most (n = 178, 87%) respondents had authorization to administer vaccines in Ontario; of these, 78% reported that they personally administer travel vaccines at their pharmacy (defined as any vaccines currently within their scope other than influenza). The most common way this service was offered was anytime by walk-in (69%), followed by appointment (24%) and during set days or hours such as a clinic day (6%); however, approximately half (54%) stated that appointment was their preferred method, with walk-in preferred by only 17% of respondents. Among the 137 individuals providing information on fees charged, 120 (88%) reported that their pharmacy charged patients a fee for this service, with CAD$20 per injection being most common (mean $18.32, SD $5.70, range $10–50). Uptake of this service appears to be low, as 94% of respondents reported administering fewer than 10 of these vaccines per month in total. Vaccines that respondents indicated they have personally administered since the expansion in scope are presented in Figure 1. Very few indicated administering any other vaccines under delegation, consisting of tetanus/diphtheria/pertussis (n = 3), measles/mumps/rubella (n = 2), and polio (n = 1).

Approximately 1 in 5 respondents (n = 56, 27%) reported that their pharmacy offers pre-travel consultation services, with walk-in being the most common model (55%) followed by an appointment basis (40%) and during clinic days (5%). By appointment is the preferred model by 62% of respondents, with only 16% preferring walk-in. Fees are charged by 35% of respondents offering this service, averaging CAD $34.17 (SD $12.81, range $20–50). As with vaccinations, pre-travel consultations are also infrequently performed, with 71% reporting performing fewer than 5 per month. 

**Figure 2:**
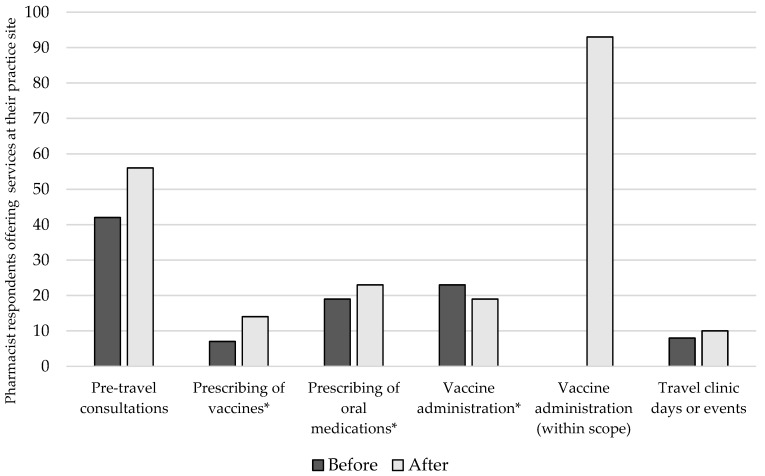
Travel-related services offered by respondents’ practice site before and after scope expansion. Asterisk indicates activities requiring delegation or medical directive from a physician or other healthcare professional. Note: * indicates activities requiring a medical directive.

As illustrated in Figure 2, increases were noted in the number of respondents whose pharmacy offered a number of travel-related services following the expansion in scope, most notably an increase in pharmacies prescribing travel vaccines under delegation (relative increase of 50%) and offering pre-travel consultation services (relative increase of 33%). After vaccine administration, offering pre-travel consultations was the most frequent service, followed by the prescribing of oral medications under delegation and vaccine administration under delegation for vaccines currently outside of scope. In terms of future plans, 71% of respondents indicated an interest in receiving their Certificate in Travel Health designation from the International Society of Travel Medicine [19] within the next 5 years, and 77% expressed an interest in either implementing or further expanding their pharmacy’s travel-related services. However, the stage of implementation was highly variable, with 38% stating they have a plan in place that they are working towards, while the remaining 62% have not established a plan.

**Figure 3:**
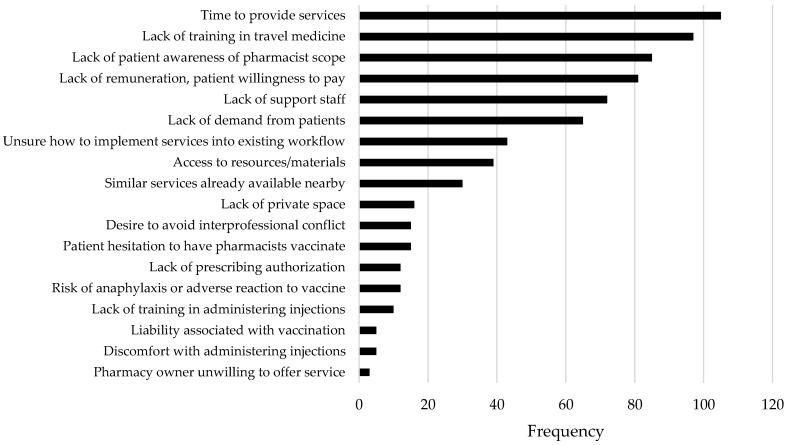
Barriers impacting ability to offer travel-related services.

**Figure 4:**
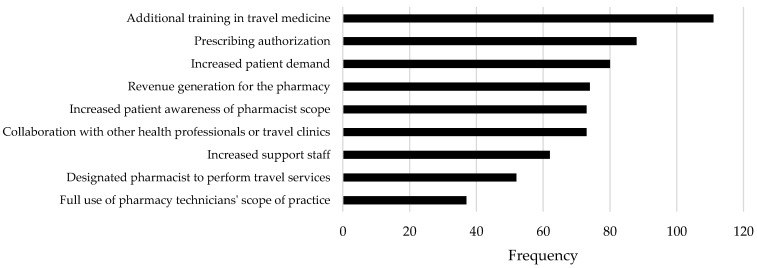
Facilitators (actual or potential) impacting ability to offer travel-related services.

Respondents reported a number of barriers impacting uptake of these services by their pharmacy and by patients as indicated in Figure 3, and actual or potential facilitators as indicated in Figure 4. Taking both reported barriers and facilitators into account, education in travel health and the authorization to prescribe appear to be key priorities to better support this service in community pharmacy practice, followed by supports related to integrating these services into existing workflow. When asked if offering these services was revenue generating for the pharmacy, responses were split, with 50.6% feeling it was revenue generating and 49.4% feeling it was not. Revenue was primarily earned through service fees charged to patients (n = 69) followed by sale of non-prescription drugs for travel purposes (n = 43), the dispensing of prescriptions for travel purposes (n = 31), and new patient recruitment as a result of offering the service (n = 26). 

### 3.2. Pharmacist Interviews

A total of six pharmacists (4 male, 2 female) from the above survey consented to participate in a semi-structured interview to further investigate pharmacy practice since the 2016 regulation change. Chain and independent practices were equally represented, as were staff pharmacists versus managers/owners. Years in practice ranged from 8–34, with one pharmacist reporting holding their Certificate in Travel Health from the International Society of Travel Medicine [21]. 

#### 3.2.1. Confidence with New Scope

While all of the pharmacists were confident in administering influenza vaccinations, pharmacists experienced varying confidence levels and fewer number of patients accessing the pharmacy for administration of the other 13 vaccines within their scope. A number of factors appeared to influence the confidence level expressed:

- **Lower demand for non-influenza vaccinations.** The proportion of pharmacy patients who had an indication for influenza vaccine (the universal immunization program in Ontario advocates for all residents without contraindications to be vaccinated) versus the other vaccines means there are fewer opportunities to exercise the expanded scope.
- **Confidence is directly related to level of exposure.** Lower exposure to administration of the new vaccinations impacted pharmacists’ confidence with both administering the vaccine and verifying its clinical appropriateness for a patient. One pharmacist practicing with a medical directive to administer non-influenza vaccinations since 2012 reported high confidence, while the others reported varying confidence based on the vaccine (e.g., more confident with herpes zoster than travel vaccines).
- **Duration of available scope.** Influenza vaccination by pharmacists in Ontario has been permitted since 2012, while additional vaccination authority was only initiated in December 2016. As such, there has been more time to gain experience with influenza vaccination, including administration and monitoring. For example, pharmacists are highly familiar with the volume, route, and adverse effects of influenza vaccination, but would need to look this information up for other vaccines. It was recognized that comfort and familiarity with the 13 new vaccines would likely increase with time as it did with influenza.

Pharmacists reported comfort with recognizing when a patient may need referral to another healthcare professional, such as last-minute travellers, those requiring yellow fever vaccination, and pediatric patients. For example, one respondent commented, “I may … refer them [the patient] to some place like … Travel Clinic because of their experience; they’re going to know which things to get onboard right away, if there’s alternative regimens that they might be able to use that I’m not comfortable suggesting” (Pharmacist 1).

Low-risk cases were considered to largely consist of healthy patients travelling to all-inclusive resorts in the Caribbean, and of those pharmacists that reported performing pre-travel consultations, most limited their practices to these low-risk cases. 

#### 3.2.2. Patient Identification and Interprofessional Collaboration

Patients requiring travel advice and other vaccinations were identified in one of three ways: (1) Patients self-identifying as needing advice; (2) Referral from other healthcare professionals; and (3) Pharmacist identification based on travel-related prescriptions presented for dispensing. Self-identified patients appeared motivated to present by an advertisement of the service or through an existing relationship with the pharmacist. Some pharmacists had collaborative relationships with other healthcare professionals who were aware of the regulation change and supported it. For example, one pharmacist described a relationship with a local nurse practitioner, saying “she has been sending a lot of her clients to us for the recommendation and then she provides a prescription“ (Pharmacist 1). However, others reported pushback from other healthcare professionals who identified this expanded scope as overlapping or competing with their services. Pharmacist 2 found this differed with physician age, stating “Some of the newer physicians were keener to utilize the pharmacist whereas some of the ones that have been around for a bit longer were more reluctant.” Pharmacist 3 similarly observed great variation, saying “Some doctors consider us as an equal and part of their practice. But some other doctors, they won’t consider or they don’t even take our recommendation.” 

#### 3.2.3. Barriers and Facilitators

Barriers emerging from the interviews generally mirrored those of the broader survey, with the exception that inability to prescribe was a more frequently cited barrier among those interviewed than among survey respondents. As one respondent noted, “Right now our biggest barrier would be prescribing of course… So we’ll gather all the information and we’ll first communicate directly with the patient, tell them what our recommendations are and whichever ones they approve or they agree with then we go through their primary care practitioner to try and get a prescription for those products” (Pharmacist 1).

**1.**  **Awareness of Pharmacists Scope:** Depending on the pharmacy and its location (e.g., co-located with a medical clinic vs. standalone), lack of awareness of the regulatory change to pharmacists’ scope was recognized among both patients and physicians, despite it being in effect for over a year at the time of the interviews. One pharmacist reported, “I don’t think [regulation change] had a big change. I don’t know if the patients are even aware about it or if it’s advertised” (Pharmacist 3).
**2.**  **Clinical Knowledge:** Travel health is a clinical area that is not routinely used daily in pharmacy practice, nor emphasized in university curricula. Pharmacists have to take it upon themselves to learn its extensive body of knowledge in order to become an expert in the field. In addition to the breadth and depth of clinical knowledge, it is also an ever-changing practice. Travel advisories, epidemics, and recommendations can change much quicker than other clinical areas (e.g., diabetes), which adds another difficulty for pharmacists to uptake travel health services. This distinction between travel health and other clinical areas was described by Pharmacist 1 as “[it’s] kind of like you need to go above and beyond what is out there to make sure you have the background information….it’s not like every day practice.”
**3.**  **Inability to Prescribe:** Pharmacists not being able to prescribe medications or vaccinations is a significant limitation to their provision of travel health services. Unlike receiving a consultation from a travel clinic, where a patient can receive their assessment, immunizations, education, and prescriptions in one appointment, patients that receive a consultation from a pharmacist must bear an additional wait time of a prescription being sent to the pharmacy from another health professional with prescribing ability. Many pharmacists expressed frustration at this limitation, with Pharmacist 1 stating “I think our profession really needs to push towards having prescribing rights for those immunizations…There is no harm in immunizing somebody, so I can’t imagine what the barrier is to getting those prescribing rights.”
**4.**  **Remuneration:** The inability to prescribe also complicates remuneration for services, since pharmacists without medical directives to prescribe rely heavily on physicians to provide prescriptions for travelling patients. Some physicians may be hesitant to do so if they offer their own pre-travel consultations for a fee or could otherwise charge for a related office visit. It is perceived that they are contributing to the consultation but without receiving a fee like the pharmacist can, which may negatively impact collaboration. One pharmacist (Pharmacist 4) noted that this can also be confusing for patients, as “the doctors want to get paid for their service for writing a prescription, in which case [my consultation is] kind of redundant. Why is the patient paying [the pharmacist] as well?”

A strong facilitator of the service mentioned by all pharmacists was the added convenience that pharmacist-provided services offers for patients. Pharmacist 5 commented “We’re open eight to eight Monday to Friday and we’re open on weekends. We close three days a year, right, so finding a time to come in and get their vaccine or whatever is not an issue for them.” While some patients were hesitant to pay an administration fee to receive the injection at a pharmacy versus at no charge from their physician’s office, Pharmacist 1 explained the return on investment as: “You can wait and go to your doctor, if you like, go book an appointment, take another day off work…. Or you can get them right here, right now and maybe wait for five minutes or ten minutes for me.”

### 3.3. Public Survey

Surveys were completed by 248 respondents (response rate 38.5%) with a mean age of 59.1 years (SD 14.0), of which 42.7%, 56.9%, and 0.4% identified as male, female, or transgendered, respectively. In total, 122 (49.2%) reported having never received a vaccine that was injected by a pharmacist. Vaccines received by a pharmacist, and the number and proportion of respondents reporting receiving each vaccine, are listed in Table 3. As influenza vaccination by pharmacists in Ontario has been in place since 2012, it is not surprising that it remains the most commonly-received vaccine by injection from a pharmacist. Interestingly, uptake of vaccines most commonly indicated for exposure related to travel were all administered by pharmacists to fewer than 5% of respondents, with the only exception being the hepatitis A&B combination vaccine at 5.6%. 

Binary logistic regression did not detect any statistically significant relationships between vaccination or non-vaccination by a pharmacist and the independent variables of age, employment status, household income, level of education, number of individuals in the household, and marital status, with the following exceptions:

- Negative relationship between increasing age and receipt of meningococcal (OR 0.847, *p* = 0.03), hepatitis B (OR 0.866, *p* = 0.004), and rabies (OR 0.839, *p* = 0.036) vaccines
- Positive association between increasing age and receipt of the herpes zoster vaccine (OR 1.05, *p* = 0.048)

### 3.4. Yellow Fever Vaccination Centres in Ontario

**Figure 5:**
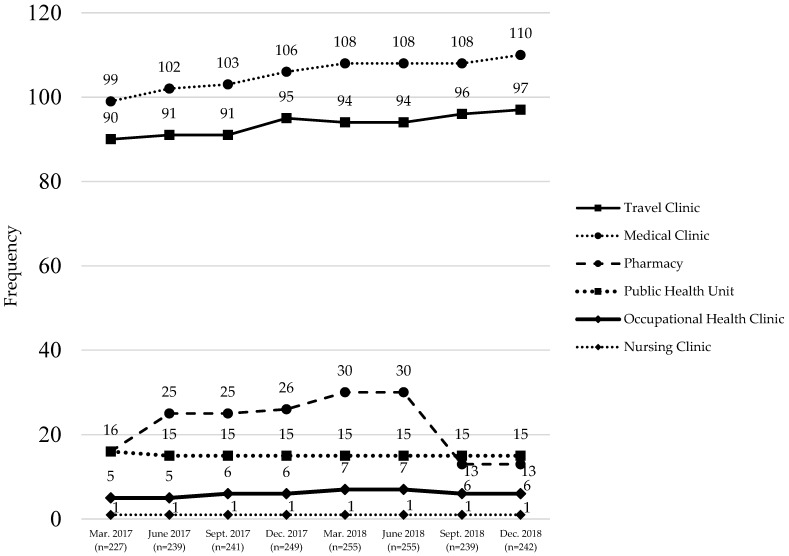
Designated Yellow Fever Vaccination Centres in Ontario, by category and date.

Prior to the expansion in pharmacist scope in December 2016, there were 220 Designated Yellow Fever Vaccination Centres in Ontario; unfortunately, data on the name of each site at this time could not be accessed; therefore, centres could not be categorized as of that date. At the first date of complete data availability, there were 227 designated centres with travel clinics and medical clinics comprising over 80% of all centres (Figure 5), followed in frequency by pharmacies and public health units, occupational health clinics, and a single nursing clinic. Small increasing trends in the number of designated sites were observed across travel clinics and general medical clinics over time, with pharmacies experiencing a near doubling between March 2017 and June 2018; however, this was immediately followed by a drop from 30 pharmacies to only 13 by September 2018 which remained consistent through December 2018. While reasons for this reduction in the number of pharmacies with designation are unavailable from the Public Health Agency of Canada, cross-referencing all pharmacies with discontinued designation against the register of licensed pharmacies in Ontario identified that all but two of these pharmacies remained licensed to operate, indicating that closure of the pharmacy was not the reason for discontinued designation status.

## 4. Discussion

Through the triangulation of multiple data sources, this study identified that the uptake of travel-related services by community pharmacies in Ontario has been generally slow, with some variation by activity and by pharmacist, since an expansion in scope to allow for the administration of travel-related vaccines in December 2016. Key challenges identified included lack of time to provide the service within existing workflow, knowledge needs related to making clinical recommendations and/or confirming the appropriateness of vaccines and prescribed drugs, and lack of prescribing authorization.

While triangulation of results from four different types of datasets was performed, limitations remain with these conclusions due to methodological design and data availability. With the pharmacist survey, the low response rate must be noted (n = 205, of over 16,000 pharmacists currently licensed in Ontario) [22], as well as under-representation of pharmacists practicing in independently-owned community pharmacies (24.9% of respondents, versus 52% of pharmacies in Ontario being independently owned), and over-representation of pharmacists with authorization to administer injections (86.8% of respondents vs. 46.8% of pharmacists in Ontario with injection administration authorization) [22,23]. Pharmacist interviews were also conducted with a small sample; however, it was felt that saturation was achieved with the responses obtained. Respondents from the public survey represented a 38.5% response rate among those contacted, but may not be representative of the overall population as the individuals invited to participate were those who had previously completed a telephone survey conducted by the Survey Research Centre at the University of Waterloo and opted-in to an online follow-up survey. Participants for the telephone survey were identified through random-digit dialing of both landline and cellular telephone numbers within the region, so individuals without access to a telephone, and individuals without internet access or comfort with internet technology, were likely excluded. Finally, as all facilities that administer the yellow fever vaccine must be registered as a Designated Yellow Fever Vaccination Centre, data on designated sites is complete; however, a worldwide shortage of yellow fever vaccine since November 2016 [24] may have negatively influenced the number of sites seeking this designation at this time.

The findings of this study are consistent with the literature on pharmacists’ uptake of new services and pharmacists’ perception of their ability to provide travel-related care. Slow uptake of new services by pharmacists in Canada was observed for activities including prescribing for medication renewals or adaptations and initial prescribing [25], prescribing for smoking cessation specifically [26], and medication reviews [27,28]. Similar trends have been observed internationally, even when remuneration is offered for the new service without having to request payment from patients [29]. Barriers and facilitators identified were consistent with those reported by pharmacists across other disease areas [30,31,32,33], in travel health specifically [14], and even by pharmacists in Ontario prior to the legislative change expanding their scope in December 2016 [16], including lack of confidence in their knowledge and skills, concerns with integrating new services into existing workflow, patient awareness of new services, and a desire for acceptance of (or enhanced collaboration with) physicians and other health professionals. We hypothesize that the clinical knowledge barrier may be more pronounced for travel health than other clinical areas due to minimal training received by many health professionals in travel health in their professional degree [34]; however, it was outside of the scope of this study to evaluate this assumption. 

## 5. Conclusions

Similarities across barriers and facilitators with previous work in travel health and other services suggest that there are systemic changes that can be made to pharmacy practice to support non-dispensing services. A movement towards appointment-based patient care models when possible, pharmacy space layout modifications to provide adequate space and privacy for consultations, investment in continuing professional development and clinical software to aid with clinical decision-making and confidence with those decisions, and advocacy efforts targeting the public to increase awareness of pharmacists’ expertise and scope will support all aspects of non-dispensing services performed in community pharmacies, with applicability to travel health and management of other clinical issues. Efforts to increase education in travel health in pharmacy curricula and the development of toolkits or standardized approaches to patient evaluation and risk management may also have a positive impact on reducing the broad variability observed related to pharmacist practice in travel health.

## Figures and Tables

**Table 1:** Vaccines that can currently be administered by authorized pharmacists in Ontario.

Vaccine	Prescription Required
Bacillus Calmette-Guérin	Yes
*Haemophilus influenzae* type B	No
Meningococcal	No
Pneumococcal	No
Typhoid	Yes
Typhoid / Hepatitis A Combination	Yes
Hepatitis A	Yes
Hepatitis B	Yes
Hepatitis A&B Combination	Yes
Herpes zoster	Yes
Human papillomavirus	No
Japanese encephalitis	Yes
Rabies	Yes
Varicella	Yes
Yellow Fever	Yes

**Table 2:** Pharmacist survey respondent characteristics.

Characteristic	Frequency (%) N = 205
**Type of community pharmacy**	
Chain	78 (38.0%)
Independent	51 (24.9%)
Banner	50 (24.4%)
Mass merchandiser	15 (7.3%)
Grocery store	10 (4.9%)
Not specified	1 (0.5%)
**Role in pharmacy**	
Staff pharmacist	93 (45.4%)
Owner	47 (22.9%)
Manager	46 (22.6%)
Relief pharmacist	18 (8.8%)
Not specified	1 (0.5%)
**Years in a community pharmacy practice**	
Less than 1	3 (1.5%)
1–5	41 (20.0%)
6–10	46 (22.4%)
11–20	46 (22.4%)
21–30	35 (17.1%)
More than 30	32 (15.6%)
Not specified	2 (1.0%)
**Average number of hours worked per week**	
Less than 8	11 (5.4%)
8–16	12 (5.9%)
17–24	16 (7.8%)
25–32	25 (12.2%)
33–40	86 (42.0%)
More than 40	51 (24.9%)
Not specified	4 (2.0%)
**Gender**	
Male	97 (47.3%)
Female	102 (49.8%)
Gender variant / non-conforming	1 (0.5%)
Not specified	5 (2.4%)
**Authorized to administer injections**	
Yes	178 (86.8%)
No	21 (10.2%)
Not specified	6 (2.9%)

**Table 3:** Vaccines reported by survey respondents as being received by injection from a pharmacist.

Vaccine	Frequency (%) (n = 248 Respondents)
Influenza	117 (47.2%)
Herpes zoster	16 (6.5%)
Pneumococcal	15 (6.0%)
Hepatitis A&B combination	14 (5.6%)
Bacillus Calmette-Guérin	8 (3.2%)
Meningococcal	8 (3.2%)
Varicella	7 (2.8%)
Typhoid	6 (2.4%)
Hepatitis B (monovalent)	6 (2.4%)
Hepatitis A (monovalent)	5 (2.0%)
Rabies	3 (1.2%)
Yellow fever	3 (1.2%)
*Haemophilus influenzae* type B	2 (0.8%)
Human papillomavirus	1 (0.4%)
Japanese encephalitis	1 (0.4%)

## Appendix A

**Table A1:** Pharmacist survey questions.

Question	Answer Options
*Screening*	
Do you currently work in a community pharmacy practice setting?	• Yes
	• No
Do you currently have an Ontario Part A license to practice pharmacy in the province?	• Yes
	• No
*Demographics*	
Which type of community pharmacy practice setting do you primarily work in?	• Independent community pharmacy
	• Community pharmacy associated with a chain
	• Community pharmacy associated with a banner
	• Community pharmacy associated with a grocery store
	• Community pharmacy associated with a mass merchandiser
	• Other (please specify)
What is your role in the community pharmacy practice setting you work in?	• Community pharmacy owner
	• Community pharmacy manager
	• Community pharmacy staff pharmacist
	• Community pharmacy relief pharmacist
Where is your community pharmacy practice setting located?	• Central East
	• Central South
	• Central West
	• East
	• North
	• South West
	• Toronto
How many years have you worked in a community pharmacy practice setting?	• Less than 1
	• 1 to 5
	• 6 to 10
	• 11 to 20
	• 21 to 30
	• More than 30
On average, how many hours per week do you work in a community pharmacy practice setting?	• Less than 8
	• 8 to 16
	• 17 to 24
	• 25 to 32
	• 33 to 40
	• More than 40
Which gender do you most identify with?	• Male
	• Female
	• Gender Variant/Non-conforming
Are you authorized to administer injections in Ontario?	• Yes
	• No
*Travel Vaccinations*	
Do you currently administer travel or travel-related vaccinations at your pharmacy?	• Yes
	• No
When and how do you currently offer travel or travel-related vaccinations at your pharmacy? Select all that apply.	• Anytime by walk-in
	• During set days/hours by walk-in (e.g., clinic days)
	• By appointment
What would be your preferred method for offering travel or travel-related vaccinations at your pharmacy?	• Anytime by walk-in
	• During set days/hours by walk-in (e.g., clinic days)
	• By appointment
	• No preference
Does your pharmacy charge a fee to patients to administer travel or travel-related vaccinations?	• Yes (please specify the fee amount)
	• No
Which of the following travel or travel-related vaccines have you personally administered since the expansion of Ontario pharmacists’ scope in December 2016? Select all that apply.	• Bacille Calmette-Guérin (BCG) (for tuberculosis)
	• Haemophilus influenza type b (Hib)
	• Hepatitis A
	• Hepatitis B
	• Combined hepatitis a and b
	• Herpes zoster (shingles)
	• Human papillomavirus (HPV)
	• Japanese encephalitis
	• Meningitis
	• Pneumococcus
	• Rabies
	• Typhoid
	• Combined typhoid and hepatitis A
	• Varicella zoster (chickenpox)
	• Yellow Fever
	• None of the above
Which other vaccines have you personally administered under delegation or a medical directive? Select all that apply.	• Diphtheria
	• IPV (poliomyelitis)
	• Measles, mumps, rubella (MMR)
	• Pertussis
	• Tetanus
	• Other (please specify)
	• None of the above
On average, how many travel or travel-related vaccinations do you administer in a month?	• Less than 5
	• 5 to 10
	• 11 to 15
	• 16 to 20
	• More than 20
*Travel Consultations*	
Do you currently perform travel consultations at your pharmacy?	• Yes
	• No
When and how do you currently offer travel consultations at your pharmacy? Select all that apply.	• Anytime by walk-in
	• During set days/hours by walk-in
	• By appointment
What would be your preferred method for offering travel consultations at your pharmacy?	• Anytime by walk-in
	• During set days/hours by walk-in
	• By appointment
	• No preference
Does your pharmacy charge a fee to patients to receive a travel consultation?	• Yes (please specify the fee amount)
	• No
On average, how many travel consultations do you complete in a month?	• Less than 5
	• 5 to 10
	• 11 to 15
	• 16 to 20
	• More than 20
*Practice Changes Related to Regulatory Expansion*	
Which travel medicine services did you offer prior to the regulatory change? Select all that apply.	• Individual travel consultations
	• Prescribing of travel or travel-related vaccines under delegation
	• Prescribing of other drugs for travel purposes (e.g., travellers’ diarrhea, altitude sickness, malaria) under delegation
	• Travel or travel-related vaccine administration under delegation
	• MedsCheck prior to travel
	• Travel medicine clinic days/events
	• Other (please specify)
	• Not applicable—I was not offering travel medicine services prior to the regulatory change
Which travel medicine services do you currently offer? Select all that apply.	• Individual travel consultations
	• Prescribing of travel or travel-related vaccines under delegation
	• Prescribing of other drugs for travel purposes (e.g., travellers’ diarrhea, altitude sickness, malaria) under delegation
	• Travel or travel-related vaccine administration under expanded scope
	• Travel or travel-related vaccine administration under delegation
	• MedsCheck prior to travel
	• Travel medicine clinic days/events
	• Other (please specify)
	• Not applicable – I am not currently offering travel medicine services
Please choose one of the following statements that best describes how your pharmacy practice has changed since the regulatory expansion with respect to the number of travel medicine services you complete on a monthly basis. (Consider all travel medicine services you offer.)	• I am completing a higher number of services
	• I am completing a lower number of services
	• I am completing about the same number of services
How does your pharmacy promote your travel-related services (vaccinations, consultations, and other related services) to patients? Select all that apply.	• In-store posters
	• Signs outside of the pharmacy
	• Newspaper/radio ads
	• Bag stuffers
	• Website
	• Social media (e.g., Facebook, Twitter)
	• Word of mouth
	• Audio ads over pharmacy's intercom system
	• Audio ads while patients are on hold on the phone with the pharmacy
	• Other (please specify)
*Practice Barriers and Facilitators*	
What have been, or would you consider to be, the primary barrier(s) to implementing travel medicine services in your community pharmacy practice? Select all that apply.	• Lack of injection training
	• Lack of travel medicine training
	• Lack of knowledge of/access to resources/materials
	• Lack of time to dedicate to travel medicine services
	• Insufficient availability of support staff (e.g., pharmacy technicians, assistants)
	• Lack of demand from patients
	• Lack of patient awareness of pharmacists’ expanded scope
	• Patients unwilling to have pharmacist vaccinate
	• Lack of remuneration
	• Unsure how to incorporate processes into daily work flow
	• Travel medicine services are already available near my place of practice
	• I am uncomfortable with administering injections
	• Religious beliefs
	• I want to avoid conflict with other professionals who can vaccinate
	• I do not want to be professionally responsible for the act of vaccination
	• I do not feel prepared to handle allergic or adverse reactions in my pharmacy
	• Lack of private space
	• Other (please specify)
Of the options you selected in the previous question, please select the ONE that you consider to be the PRIMARY barrier to implementing travel medicine services in your community pharmacy practice.	• Lack of injection training
	• Lack of travel medicine training
	• Lack of knowledge of/access to resources/materials
	• Lack of time to dedicate to travel medicine services
	• Insufficient availability of support staff (e.g., pharmacy technicians, assistants)
	• Lack of demand from patients
	• Lack of patient awareness of pharmacists’ expanded scope
	• Patients unwilling to have pharmacist vaccinate
	• Lack of remuneration
	• Unsure how to incorporate processes into daily work flow
	• Travel medicine services are already available near my place of practice
	• I am uncomfortable with administering injections
	• Religious beliefs
	• I want to avoid conflict with other professionals who can vaccinate
	• I do not want to be professionally responsible for the act of vaccination
	• I do not feel prepared to handle allergic or adverse reactions in my pharmacy
	• Lack of private space
	• Other (please specify)
What have been, or would you consider to be, the primary facilitator(s) for the implementation of travel medicine services in your community pharmacy practice? Select all that apply.	• Completion of additional training in immunization or travel medicine
	• Collaboration with other healthcare professionals or health clinics
	• Increased patient demand
	• Increased awareness of pharmacists’ expanded scope
	• Increased support staff hours
	• Increased use of pharmacy technicians’ scope of practice
	• Designated travel medicine services pharmacist
	• Pharmacists’ ability to prescribe travel vaccines or travel-related medicine
	• Revenue generation
	• Other (please specify)
Of the options you selected in the previous question, please select the ONE that you consider to be the PRIMARY facilitator to implementing travel medicine services in your community pharmacy practice.	• Completion of additional training in immunization or travel medicine
	• Collaboration with other healthcare professionals or health clinics
	• Increased patient demand
	• Increased awareness of pharmacists’ expanded scope
	• Increased support staff hours
	• Increased use of pharmacy technicians’ scope of practice
	• Designated travel medicine services pharmacist
	• Pharmacists’ ability to prescribe travel vaccines or travel-related medicine
	• Revenue generation
	• Other (please specify)
Do you find your pharmacy’s travel medicine service(s) to be revenue generating?	• Yes
	• No
What do you feel primarily contributes to the profitability of your service(s). Select all that apply.	• A fee-for-service is charged to patients
	• More people are becoming patients at my pharmacy because of the travel medicine services I/we offer
	• More travel-related OTC sales after counselling are occurring
	• More patients are bringing in their travel-related prescriptions because of the travel medicine service I/we offer
	• Efficiencies (through optimal use of staff, materials/resources used, implementation of processes, etc.) have been created as a result of implementing travel medicine services
	• Not applicable – a business evaluation of this service has not yet been conducted by our pharmacy
	• Other (please specify)
*Education Needs and Preferences*	
Which areas of additional travel medicine-related education would facilitate the implementation or improve the quality of pharmacist-led travel medicine services in your community pharmacy? Select all that apply.	• Processes for completing a pre-travel assessment
	• Pre-travel assessment knowledge based on geographical location(s) during travel
	• Pre-travel assessment knowledge based on activity/activities during travel
	• Pre-travel assessment knowledge based on patients’ health status, comorbidities, and special patient populations
	• Travel-related diseases
	• Travel-related vaccines
	• Travel-related prescription medication use
	• Travel-related self-care and non-prescription drug measures
	• Other travel health risks
	• Other (please specify)
	• Not applicable – I feel confident in my travel medicine-related knowledge and training background
With respect to acquiring further travel medicine-related education, which forms of education would be most appealing to you? Select up to 5 options.	• Self-directed online continuing education course of <2 h duration
	• Self-directed online continuing education course of 2+ h duration
	• Webinar
	• In-person or live continuing education course (half day)
	• In-person or live continuing education course (full day)
	• Downloadable clinical practice guidelines on travel medicine topics
	• Downloadable or printable educational materials (e.g., booklets)
	• Mobile app or online resources
	• Self-assessment tools
	• Diploma/certificate level training in travel medicine
	• Other (please specify)
Would you be interested in pursuing a Certificate in Travel Health designation from the International Society of Travel Medicine in the next 5 years?	• Yes • No • Unsure
*Future Plans and Advocacy*	
Which of the following statements best describes your future plans with respect to offering travel medicine services in your pharmacy?	• Already implementing, and not currently working on a plan to further expand
	• Already implementing, and wanting/working on a plan to further expand
	• Intend to implement, and currently working on a plan
	• Interested in implementing, but not currently working on a plan
	• No intention to implement in the near future
In addition to allowing additional vaccines to be administered, the regulatory changes under the *Pharmacy Act* also now allow for injection trained pharmacy students and interns to administer the same vaccines. Which of the following statements best describes how your pharmacy practice has changed since the regulatory change with respect to pharmacy students and interns administering vaccines?	• My pharmacy now has pharmacy students and/or interns administering vaccines
	• My pharmacy does not have pharmacy students or interns administering vaccines and does not intend to have them administering vaccines
	• My pharmacy does not have pharmacy students or interns administering vaccines but is considering having them administer vaccines in the future
	• Unsure
	• Not applicable – vaccines are not administered at my pharmacy
Would you want OPA to advocate for pharmacists’ ability to prescribe travel and travel-related vaccines?	• Yes
	• No
	• Unsure
Would you want OPA to advocate for pharmacists’ ability to prescribe travel-related drugs (e.g., travellers’ diarrhea, malaria, altitude sickness, etc.)?	• Yes
	• No
	• Unsure

OPA, Ontario Pharmacists Association; OTC, over-the-counter.

## Appendix B

Semi-structured interview guide for pharmacists.

Let’s start with some questions about your current practice:

- Please describe your pharmacy’s patient population *(Prompt if needed: for example, age, socioeconomic status, health conditions)*
- Please describe your relationships with other healthcare providers in your community and their perception of your role as a patient care provider.
- Is there a travel clinic or another health professional that currently provides travel health services in your community? Does this effect the types of services you offer?
- Can you explain your experience, if any, with administering influenza vaccines in your pharmacy? *(Prompt if needed: How long have you been doing this? How many patients have you administered to? What is your comfort level with this?)*
- Can you explain your experience, if any, with administering other vaccines that are generally **not** used for travel, such as pneumococcal, zoster, or HPV vaccine? *(Prompt if needed: How long have you been doing this? How many patients have you administered to? What is your comfort level with this?)*

Tell me about your experiences with travel health services in your practice? (Prompt if needed to discuss both administering travel vaccines and performing consultations. A consultation is an individualized assessment of a person’s health risks while travelling, considering patient-specific, destination-specific, and activity-specific factors).

What impact, if any, has the regulatory change to expand the list of vaccinations that pharmacists can administer had on your services? Why?

**IF they have experience** **with administering travel vaccines in their pharmacy:**

Regarding administering travel vaccines, such as those for hepatitis A or B, typhoid, Japanese encephalitis, or yellow fever:

- Describe your process for administering these vaccines and how it fits within your workflow.
- How do you identify eligible patients?
- Are there any clinical or non-clinical circumstances where you would not administer travel vaccinations to a patient who requested it? *(Prompt if needed: What are these, and why?)*
- Describe your process for documentation. *(Prompt if needed: Do you inform the patient’s primary care provider that you administered these vaccines?)*
- Do you provide patients with any written educational materials about these vaccines? If so, which resources do you provide? *(Prompt if needed: Information from website online, pamphlet provided by vaccine manufacturer, written information developed in-house)*
- Tell me about your rationale for either charging, or not charging, a fee for administering these vaccines. **IF a fee is charged:** How have your patients responded to being asked to pay for this service?

**IF they have experience with** **performing travel consultations in their pharmacy:**

Regarding providing travel consultations:

- Describe your process for providing this service and how it fits within your workflow.
- How do you identify eligible patients?
- Are there any clinical or non-clinical circumstances where you would refer a patient to another healthcare professional for a consultation? *(Prompt if needed: What are these, and why?)*
- Describe your process for documentation. *(Prompt if needed: Do you inform the patient’s primary care provider that you performed this consultation?)*
- Do you provide patients with any written educational materials about travel health? If so, which resources do you provide? *(Prompt if needed: Information from website online, pamphlet provided by vaccine manufacturer, written information developed in-house)*
- Tell me about your rationale for either charging, or not charging, a fee for this consultation? **IF a fee is charged:** How have your patients responded to being asked to pay for this service?

**For any pharmacists** **with experience with either or both of the activities above:**

Let’s talk about your overall experience as a travel health care provider:

- Tell me about facilitators and barriers you have experienced in delivering travel health services to your patients? What strategies have you tried to make it easier for you to provide this care in practice?
- Please describe for me your level of confidence with providing travel health services?
- What feedback, if any, have you received from your patients?
- What feedback, if any, have your received from other healthcare providers in your community?

**For pharmacists that report** **NOT administering travel vaccines and NOT providing travel consultations:**

- Under what clinical circumstances would you consider providing your patients with:
  ◦ Travel vaccine administration?
  ◦ Travel consultations and education?
- What are some reasons why you have not offered these services in your pharmacy? *(Prompt if needed: Some examples might be staffing, management support, need for more training or confidence, or do you feel this is outside of the scope of practice for pharmacists?)*
- What are some supports that would help you to offer these types of services?
- If your pharmacy were to start providing travel health services, would you charge a fee for administering vaccines or providing travel consultations? Why or why not?

## Appendix C

Waterloo Region Area Survey questions.

**Demographic** **Questions:**

Please select your gender:

- Male
- Female
- Transgendered

What is your current employment status? **(SELECT ONE ONLY)**

- Full-time
- Part-time
- Retired
- Unemployed
- Student
- Homemaker
- Other (Please specify: _____________)

What is your current household income before taxes? **(SELECT ONE ONLY)**

- Less than $20,000
- $20,000 to less than $50,000
- $50,000 to less than $80,000
- $80,000 to less than $100,000
- $100,000 or more

What is the highest level of formal education that you have completed? **(SELECT ONE ONLY)**

- Grade school
- High school
- College or trade apprenticeship
- University degree
- Other (Please specify: _____________)

Not including yourself, how many people live in your household? Please include all adults, other than yourself, and any children that live with you all or most of the time.

At present, are you married, living with a partner, widowed, divorced, separated, or have you never been married? **(SELECT ONE ONLY)**

- Married
- Living with partner/common-law
- Widowed
- Divorced or separated
- Never married

**Pharmacist-Administered** **Vaccines Question:**

Please indicate which vaccines, if any, you have **ever** received **by injection** by a **pharmacist:** (Please select all that apply)

- Influenza (flu) vaccine
- Bacille Calmette-Guérin (tuberculosis) vaccine
- Haemophilus Influenzae type b (Hib) vaccine
- Meningococcal (meningitis) vaccine
- Pneumococcal (pneumonia) vaccine
- Typhoid vaccine
- Hepatitis A vaccine
- Hepatitis B vaccine
- Hepatitis A and B combined vaccines (e.g., Twinrix^®^)
- Herpes Zoster (shingles) vaccine
- Human Papillomavirus (HPV, cervical cancer) vaccine
- Japanese Encephalitis vaccine
- Rabies vaccine
- Varicella (chickenpox) vaccine
- Yellow Fever vaccine
- I have never received a vaccine that was injected by a pharmacist.
